# Synthesis and Characterization of Paclitaxel-Loaded Silver Nanoparticles: Evaluation of Cytotoxic Effects and Antimicrobial Activity

**DOI:** 10.1155/2024/9916187

**Published:** 2024-02-13

**Authors:** Tutku Tunç, Ceylan Hepokur, Afşin Kari̇per

**Affiliations:** ^1^Department of Pharmaceutical Microbiology, Faculty of Pharmacy, Sivas Cumhuriyet University, Sivas, Türkiye; ^2^Department of Biochemistry, Faculty of Pharmacy, Sivas Cumhuriyet University, Sivas, Türkiye; ^3^Department Mathematics and Science Education, Faculty of Education, Erciyes University, Kayseri, Türkiye

## Abstract

Carrier system therapies based on combining cancer drugs with nanoparticles have been reported to control tumor growth and significantly reduce the side effects of cancer drugs. We thought that paclitaxel-loaded silver nanoparticles (AgNPs-PTX) were the right carrier to target cancer cells. We also carried out antimicrobial activity experiments as systems formed with nanoparticles have been shown to have antimicrobial activity. In our study, we used easy-to-synthesize and low-cost silver nanoparticles (AgNPs) with biocatalytic and photocatalytic advantages as drug carriers. We investigated the antiproliferative activities of silver nanoparticles synthesized by adding paclitaxel on MCF-7 (breast adenocarcinoma cell line), A549 (lung carcinoma cell line), C6 (brain glioma cell line) cells, and healthy WI-38 (fibroblast normal cell line) cell lines and their antimicrobial activities on 10 different microorganisms. The synthesized AgNPs and AgNPs-PTX were characterized by dynamic light scattering (DLS), scanning transmission electron microscopy, UV-visible spectroscopy, Fourier transform infrared spectroscopy, and X-ray spectroscopy. The nanoparticles were spherical in shape, with AgNPs ranging in size from 2.32 to 5.6 nm and AgNPs-PTXs from 24.36 to 58.77 nm. AgNPs demonstrated well stability of −47.3 mV, and AgNPs-PTX showed good stability of −25.4 mV. The antiproliferative effects of the synthesized nanoparticles were determined by XTT (tetrazolium dye; 2,3-bis-(2-methoxy-4-nitro-5-sulfenyl)-(2H)-tetrazolium-5-carboxanilide), and the proapoptotic effects were determined by annexin V/propidium iodide (PI) staining. The effect of AgNPs-PTX was more effective, and anticancer activity was higher than PTX in all cell lines. When selectivity indices were calculated, AgNPs-PTX was more selective in the A549 cell line (SI value 6.53 *μ*g/mL). AgNPs-PTX was determined to increase apoptosis cells by inducing DNA fragmentation. To determine the antimicrobial activity, the MIC (minimum inhibitory concentration) test was performed using 8 different bacteria and 2 different fungi. Seven of the 10 microorganisms tested exhibited high antimicrobial activity according to the MIC ≤100 *μ*g/mL standard, reaching MIC values below 100 *μ*g/mL and 100 *μ*g/mL for both AgNPs and AgNPs-PTX compared to reference sources. Compared to standard antibiotics, AgNPs-PTX was highly effective against 4 microorganisms.

## 1. Introduction

Nanotechnology has recently advanced quickly and begun to be applied in a variety of industries, including medicine. Nanotechnology offers new technologies for the production of nanomaterials at the scale of 1–100 nm, which are especially used in the treatment of infections and cancer cells caused by drug-resistant bacteria [1]. Silver nanoparticles (AgNPs) are increasingly being investigated in the medical field due to their unique physical, chemical, and optical properties [2]. Silver has been used for many years as an important antibacterial agent [3]. Recent studies have demonstrated that silver nanoparticles have intrinsic antibacterial and anticancer effects via a variety of pathways. The possibility of combining the pharmacological action of anticancer drugs with the anticancer intrinsic properties of AgNPs may be useful for the treatment of tumors that have ceased to respond to chemotherapy or radiotherapy. The ability of AgNPs to be used to deliver anticancer drugs to a tumor microenvironment is a promising treatment approach for cancer [4].

Cancer is the global cause of death in both high-income and middle-income countries [5]. According to the Global Cancer Observatory (GLOBOCAN), the five cancers with the highest death rates worldwide are lung cancer (18%), colorectal cancer (9.4%), liver cancer (8.3%), stomach cancer (7.7%), and breast cancer (6.9%) [6]. Lung cancer (LC) is the foremost cause of cancer-related fatalities globally [7]. Treatment of LC and its subtypes, small-cell lung cancer (SCLC) and non-small cell lung cancer (NSCLC), depends on chemotherapy, radiotherapy, immunotherapy, and targeted treatment mechanisms. On the other hand, despite their potential efficacy, their application leads to serious side effects, reduced efficacy, development of drug resistance, and the potential for relapse. Therefore, new and safe therapeutic alternatives need to be evaluated [8, 9].

One of the most effective anticancer drugs, paclitaxel (PTX), is used to treat lung, breast, and ovarian cancers; however, it has limited therapeutic applicability due to its poor solubility [10]. Recently, various drug delivery methods, including polymer nanoparticles, have been investigated to improve the solubility of PTX and prevent negative side effects. Polymer nanoparticles have drawn a lot of attention among these new medication delivery methods [11].

Chemotherapeutic agents and nanoparticle combinations have shown great interest in cancer therapy. Combined treatment of anticancer drugs and nanoparticles at low doses promotes synergy through different mechanisms of action and suppresses drug resistance. Combination therapy mediated by nanoparticles improves pharmacokinetics, decreases unfavorable side effects, and promotes anticancer activity [12]. Moreover, combination therapy can prevent toxic effects on normal cells and combat the expected acquired resistance or minimize the likelihood of developing drug resistance [13, 14].

AgNPs may make good prospective combination partners, as it has recently been revealed that they alter Pgp (permeability glycoprotein) activity, improving the effectiveness of chemotherapy against multidrug-resistant cancer cells. Additionally, the production of double-stranded DNA breaks with chromosomal instability, which facilitates the start of the apoptotic process and contributes to AgNPs' genotoxicity [15, 16].

According to our research, the produced silver nanoparticle with paclitaxel added was the ideal vehicle for concentrating on cancer cells. Therefore, we used AgNPs, which have biocatalytic and photocatalytic advantages, easy to synthesize, and low cost, as drug carriers in our study. We aimed to investigate their antiproliferative effects on MCF-7 (breast adenocarcinoma cell line), A-549 (lung carcinoma cell line), C6 (brain glioma cell line), and noncancerous WI-38 (fibroblast normal cell line) cells. Additionally, another aim of our study was to determine the antimicrobial activity of AgNPs and AgNPs-PTX against bacteria and yeasts.

## 2. Materials and Methods

### 2.1. Materials

Silver nitrate (Ag(NO_3_)), sodium boron hydride (NaBH_4_), trisodium citrate, dimethylsulfoxide (DMSO), and XTT cell proliferation assay kit were purchased from Sigma (St. Louis, USA). Dulbecco's Modified Eagle's Medium (DMEM), penicillin-streptomycin, trypsin, and fetal bovine serum (FBS) were purchased from Gibco (USA). 20x phosphate-buffered saline (PBS) was purchased from Thermo Fisher (USA), cation-adjusted Mueller-Hinton broth (CAMHB) and 2,3,5-triphenyltetrazolium chloride (TTC) were purchased from Merck (Germany), and annexin V/PI assay kit BD (USA) and paclitaxel were purchased from Sandoz (Germany).

### 2.2. Synthesis and Characterization of Silver Nanoparticles and Paclitaxel-Loaded Silver Nanoparticles

Stock solutions of 2 × 10^–3^ M Ag(NO_3_), 8.6 × 10^–3^ M trisodium citrate, and 4 × 10^–3^ M sodium boron hydride were prepared. Then, 12 mL trisodium citrate, 12 mL sodium boron hydride, and 24 mL pure water were mixed at 60°C for 30 minutes. At that time, the temperature was raised to 90°C, and 48 mL of silver nitrate was added to the stock solution. A few minutes after the yellow color change was seen, the reaction was stopped, and the AgNPs stock solution was stored in a dark cool environment. 10 mg/10 mL PTX stock solution was prepared. The mixture of 1 mL PTX stock solution and 1 mL AgNPs stock solutions was sonicated with an ultrasonication instrument for 10 seconds. (the solution pH is 7, approximately.) Then, the mixing is waited in the dark for at least 30 minutes.

Zetasizer measurements were made with dynamic light scattering (DLS) measurements using the Zetasizer Nano ZS, which uses a 4 mW He-Ne laser operating at a wavelength of 633 nm and a detection angle of 173° at room temperature. Distilled water was used as the reference liquid. In the measurements, both dimensional and surface load analyses were made.

First of all, the samples were dropped on amorphous lamella glass substrates. These base materials were previously washed with detergent and passed through plenty of distilled water. The samples were left to dry in a clean room for 1 night at room temperature and under normal atmospheric conditions. These samples were then used for STEM-EDX analyses. The surface properties of the powders were examined using a Gemini 500 computer-controlled digital transmission electron microscopy (STEM). Quantitative elemental analysis was performed with an EDX spectrometer attached to STEM.

The absorbance measurements of the nanoparticles were determined by Hach Lange 500 Spectrophotometer at room temperature by placing an uncoated identical commercial glass substrate in the reference beam. The optical spectrum of thin films was recorded in the wavelength range of 300–1100 nm.

Fourier transform infrared spectroscopy (FTIR) studies were taken with the BRUKER ALPHA instrument at a resolution of 4 cm × 1 in diffuse reflection mode. The measurement of each sample was recorded after 10 scans. The wavelength range for FTIR is 450–4000 cm^−1^.

### 2.3. Cell Culture

MCF-7 (breast adenocarcinoma cell line), A-549 (lung carcinoma cell line), C6 (brain glioma cell line), and noncancerous WI-38 (fibroblast normal cell line) cell lines were supplied by the American Type Culture Collection (Manassas, VA). All cells were cultured in DMEM supplemented with 2 mM L-glutamine, 10% heat-inactivated FBS, 1% penicillin, and streptomycin, with 5% CO_2_ supply at 37°C for 48 hours [17]. The cells were removed by a solution of 0.05% trypsin-0.02% EDTA when the confluence was 90% reached.

XTT test was used to investigate cell viability. A flat-bottomed 96-well cell culture plate was used, and cells were seeded into each well at a density of 10^4^ (200 *μ*L medium per well). After 24 hours, cells were treated with different concentrations of AgNPs-PTX and PTX (100-50-25-12.5-6.25-3.125-1.56 *μ*g/ml) and incubated for 24 hours. DMSO was used as the negative control. DMSO was added to the negative control wells at 2%. Next, 10 *μ*l of XTT solution was added to each well, and the absorbance was measured at 450 nm using a microplate reader (SPECTROstar Nano Microplate Reader, BMG LabTech Instruments, Inc., Ortenberg, GERMANY) [18]. Cell viability was calculated according to the following formula:

(1) $$\begin{array}{c} \text{Cell}\;\text{viability}\left(\%\right)=\left[\frac{\left(A_{s}-A_{b}\right)}{\left(A_{c}-A_{b}\right)}\right]\times 100, \end{array}$$

where *A*_*s*_ is absorbance sample, *A*_*b*_ is absorbance blank, and *A*_*c*_ is absorbance viable cell (control), and IC_50_ values were calculated by using XTT analysis [19].

The calculated cell viability against the concentration graph (SigmaPlot 12.0 program) was plotted. IC_50_ value was found by using the plotted graph.

To examine the cell morphology, 1 × 10^5^ cells were inoculated into 96 plates. 15 *μ*g/mL AgNPs-PTX and PTX were applied to the cells and incubated for 24 hours. After the medium was emptied, cells were fixed using 70% ethanol and their images were taken with a ZEISS Axio inverted microscope.

### 2.4. Apoptosis Analysis (Annexin/PI Flow Cytometry Analysis)

To quantify apoptotic cells, annexin V/propidium iodide (PI) staining was performed using A549 and healthy WI-38 cells with the highest cytotoxic activity. Flow cytometric analysis of AgNPs-PTX and paclitaxel at IC_50_ concentrations were analyzed using annexin V/PI commercial kits. To determine apoptotic cells, A-549 and WI-38 cells (1 × 10^5^) were grown in a 6-well culture plate. IC_50_ values of AgNPs-PTX and paclitaxel were processed in A549 and WI-38 cells for 24 hours. At the end of this period, the contents of the plate were removed, and the cells were washed with PBS, treated with trypsin-EDTA, and then centrifuged at 800 rpm for 8 minutes. The collected cells were counted with trypan blue. Then, 5 *μ*L annexin V and 5 *μ*L PI were added to the medium. Cells were stored at room temperature and in the dark for 15 minutes. After the time was over 400 *μ*L, the binding buffer was added on ice, and flow cytometry measurements were analyzed (emission: 530 nm and excitation: 488 nm) [19, 20].

### 2.5. Antimicrobial Assay

Microorganisms to be used in the MIC test, *Escherichia coli* (ATCC 25922), *Klebsiella pneumoniae* (ATCC 13883), *Acinetobacter baumannii* (ATCC 17978), *Pseudomonas aeruginosa* (ATCC 27853), *Staphylococcus aureus* (ATCC 29213), *Methicillin-resistant Staphylococcus aureus* (MRSA) (ATCC 43300), *Enterococcus faecalis* (ATCC 29212), *Bacillus cereus* (ATCC 11778), *Candida albicans* (ATCC 10231), and *Candida tropicalis* (ATCC 4563) were obtained from the American Type Culture Collection (Rockville, MD, United States). Bacteria were passaged in brain heart infusion broth (BHI) (Merck, Germany), and fungi were passaged in Sabouraud Dextrose Broth (SDB) (Merck, Germany) media after incubation at 37°C for 24 hours.

The antimicrobial activity of AgNPs and AgNPs-PTX was determined using the MIC (minimum inhibition concentration) method. 10 *μ*l of synthesized material (100 *μ*g/ml) was added to the first row of U-bottomed 96-well plates. Then, two-fold serial dilutions were made for a total of 10 concentrations. 50 *μ*l of microorganism culture (5 × 10^5^ CFU/mL for bacteria and 0.5–2.5 × 10^3^ CFU/mL for yeasts) adjusted to McFarland turbidity of 0.5 was seeded to the wells. 100 *μ*l CAMHB was added to the sterility control section of the plate. 50 *μ*l CAMHB +50 *μ*l microorganism culture was plated to the last rank of the plate for growth control. The standard antibiotics to which the microorganisms were susceptible were added 50 *μ*l to the microorganism cultures in the bottom rank of the plates [21, 22]. Bacterial plates were incubated at 37°C for 24 hours and yeast plates for 48 hours. After incubation, 2 mg/ml of 2,3,5-triphenyltetrazolium chloride (TTC) (Merck, Germany) solution was added to each well to detect microorganism growth on the plates. The plates were incubated at 37°C for 2 hours. The first well with a reduction in the color of the formazan indicating the viability of the microorganisms was assessed as the MIC concentration. MIC results according to reference sources. It was evaluated as effective (MIC < 100 *μ*g/mL), moderate (100 < MIC ≤ 625 *μ*g/mL), and weak (MIC > 625 *μ*g/mL) [23, 24]. Evaluation was also determined according to the MIC results of standard antibiotics [25]. The experiment was repeated three times, and the standard deviation was found to be zero.

### 2.6. Statistical Analysis

The results were analyzed using SPSS software version 20 (IBM Statistics, USA). Differences in measured properties of the groups were tested using a one-way analysis of variance (ANOVA). The significance level was set as *p* ≤ 0.05.

## 3. Results and Discussion

Nanomedicine uses nanomaterials and applies nanotechnology to the diagnosis, treatment, and prevention of diseases. These include applications of medical nanosensors, biochips, needleless injectors, insulin pumps, and nanoparticles as drug carrier systems [26]. Drug carrier systems and nanostructures can overcome the stability and solubility problems of anticancer drugs. The drug is protected from proteases and other enzymatic degradation, thereby prolonging the drug's half-life in the systemic bloodstream; these benefits include improving drug targeting and delivery, helping to potentially release drugs at targeted cancer sites, and creating multiple drug carriers to help reduce drug resistance [27, 28].

### 3.1. Synthesis and Characterization of AgNPs and AgNPs-PTX

DLS results are given in Table 1. While the average size of the synthesized AgNPs was 2.32 nm, the particle distribution was determined at 5.61 nm in the nanosizer (Table 1).

When the paclitaxel drug was attached to AgNPs, the average particle size increased to 34.56 nm, and the largest particle was detected at 58.77 nm. However, the average particle size of the AgNPs increased to 2.32 nm, and the largest particle was detected at 5.61 nm. This means that when the paclitaxel drug was attached to AgNPs, the average particle size increased to 58.77 nm. While the PDI value of AgNPs was 0.200, this value increased to 0.535 when the drug was bound. This indicates that even though the drug is bound to AgNPs, the nanoparticles are still stable according to the data of the device. Also, this indicates to growth of the particles with physical or chemical bonding. While the surface charge of the synthesized AgNPs was −47.3 mV, it increased to −25.4 mV when the drug was bound. The conductivity of the solution increased from 0.314 mS/cm to 3.39 mS/cm for AgNPs. Although the pure water environment does not simulate the human body, the increase in conductivity suggests that with the increase of the surface charge, drug-bound nanoparticles will increase the diffusion of cancer cells. Nanodrugs with negative surface charge are effective in cancer cells [29]. When the paclitaxel drug was attached to AgNPs, the particle size, PDI, zeta potential, and conductivity of particles were increased. AgNPs surface area is negative and reduced when added with regents and stabilisers. When the paclitaxel drug is attached to AgNPs, drug-AgNPs surface area approaches positive. Paclitaxel molecules coat and close the surface area of the AgNPs. This is the reason for the increase of the zeta potential and the growth of the particle size. In this manner, the PDI is increased with the particle size.

**Figure 1:**
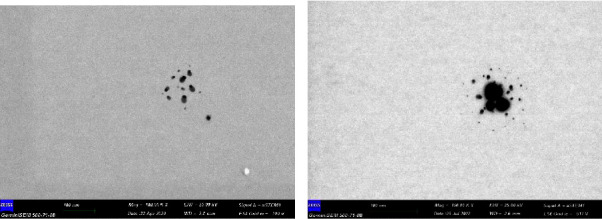
(a) STEM image of AgNPs and (b) STEM image of AgNPs-PTX.

STEM images are given in Figure 1.

Considering the 100 nm scale in STEM images, it is seen that AgNPs are 10 nm and below, while drug-bound AgNPs-PTX particles have particle sizes around 10–50 nm. STEM images confirm DLS results.

**Figure 2:**
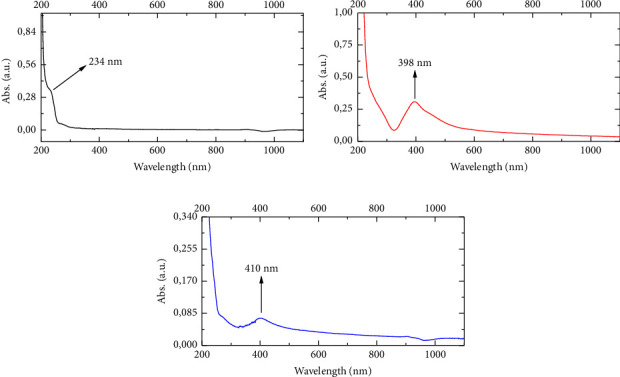
The UV-VIS spectrum of (a) paclitaxel, (b) AgNPs, and (c) AgNPs-PTX.

The UV-VIS spectrum of paclitaxel (Figure 2(a)), AgNPs (Figure 2(b)), and AgNPs-PTX (Figure 2(c)) is given in Figure 2. As expected, PTX dissolved in an aqueous medium gave a specific absorption band at 234 nm, in Figure 2(a). This is due to *n*-*σ*^*∗*^, *σ*-*σ*^*∗*^, and *π*-*π*^*∗*^ electronic transitions in the amine, hydroxyl, and C=C groups in the structure of paclitaxel. Especially, due to the aromatic ring structures, *π*-*π*^*∗*^ electronic transitions are widespread [30, 31].

In the spectrum of AgNPs, a specific absorption band specific to AgNPs is observed at 398 nm, as shown in Figure 2(b). This band, which should be seen at 400 nm, has shifted to 398 nm [32]. The reason for this is that the nanoparticle size shifts to a lower wavelength as the particle size decreases with the quantum dot effect [33]. When the drug was bound to AgNPs, the maximum absorption band was observed at 410 nm, as shown in Figure 2(c). As the particle size increased, the absorption band shifted to a higher wavelength. This is an indication that the drug also chemically binds to AgNPs [29].

**Figure 3:**
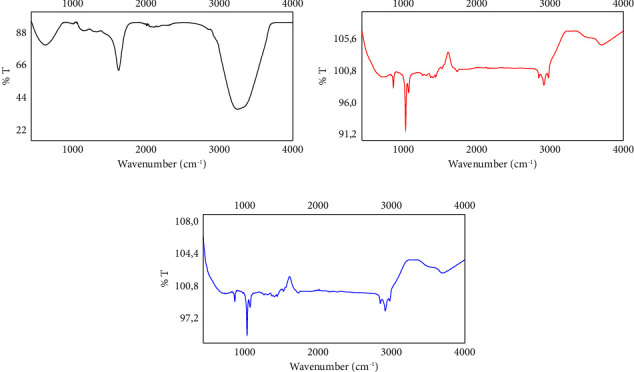
The FTIR spectrum of (a) AgNPs, (b) PTX, and (c) AgNPs-PTX.

FTIR spectrum of AgNPs (Figure 3(a)), PTX (Figure 3(b)), and AgNPs-PTX (Figure 3(c)) is shown in Figure 3. Vibration peaks of –OH and –NH groups are observed at 3252 cm^−1^. While the peaks of –C=O stretching vibrations are observed at 1631 cm^−1^, in general, the specific characteristic peak of the nanoparticles was observed at 631 cm^−1^. As expected in the FTIR spectrum of paclitaxel, bond vibration bands belonging to –OH and –NH functional groups were observed at 3705 cm^−1^, while symmetric vibration bands belonging to aliphatic groups were also detected at 2997-2921-2860 cm^−1^. At 1757 cm^−1^, –NH and -NH2 bending vibration contributions were determined. A vibration band of –CO ether at 1041 cm^−1^ and specific vibration bands of aromatic –C=C group at 882 cm^−1^ were detected [34, 35]. However, when the drug is bound to AgNPs, the vibration band of the –OH functional groups weakened a little but shifted to 3600 cm^−1^. This is an indication of the chemical interaction between oxygen and Ag in the –OH groups of paclitaxel. This FTIR spectrum also supports the UV-VIS spectrum results in terms of chemical bonding. Apart from this, no change was observed in other vibrational bands of drug-bound AgNPs.

**Figure 4:**
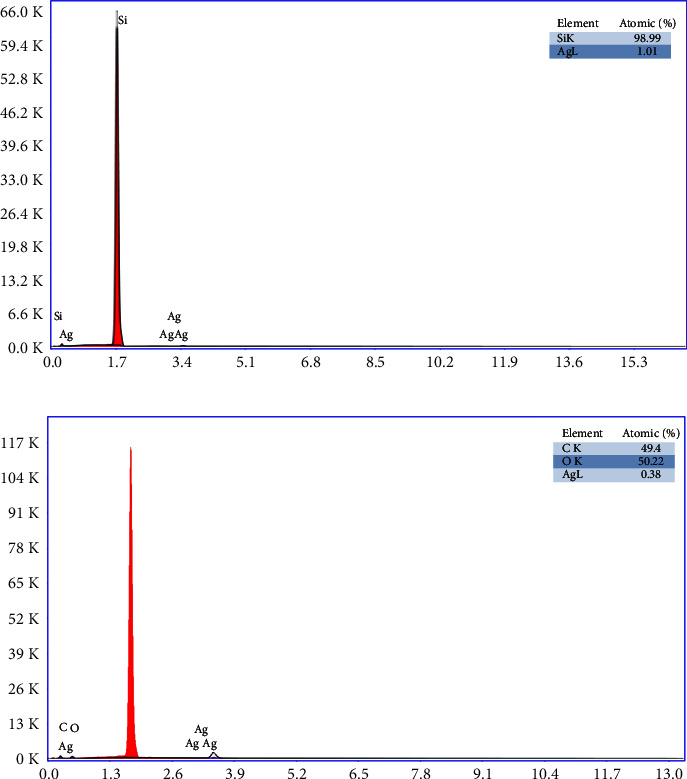
(a) EDX analysis of AgNPs and (b) EDX analysis of AgNPs-PTX.

EDX analyses of AgNPs and AgNPs-PTX particles are given in Figure 4. Of course, the elements that do not affect the work are neglected here. According to the results of the analysis, 1.01% Ag was detected in AgNPs, while this rate decreased to 0.38% with the effect of drug-bound AgNPs in the drug. In addition, drug-bound AgNPs were found to be 49.4% carbon and 50.22% oxygen, which is one of the specific elements of the drug. At the same time, the inability to detect nitrogen is a surprising result.

### 3.2. Cytotoxicity Analysis

Three different cancer cell lines and one healthy cell were used in this study. The viability values obtained as a result of the XTT method, and the IC_50_ values were calculated (Table 2).

Table 2 indicated that the AgNPs molecule did not cause significant cytotoxicity in any cell group. This result confirms that the synthesized nanoparticles are harmless and can be used in cancer treatment. Furthermore, AgNPs-PTX was found to be more effective than paclitaxel alone in all cancer cells. Statistically, AgNPs-PTX showed a significant difference against healthy cells for all cancer cells (*p* < 0.05). The selectivity index (SI) value was calculated to find the cell line that showed the highest effect among the cells (Table 3).

To evaluate any anticancer activity of a product, its cytotoxicity against noncancerous cell lines must be established to calculate its SI value. Ideally, the drug should be able to kill cancer cells but should not affect normal cells. A low SI value (<1) means that the sample may be toxic and cannot be used as a drug. If the computed SI value is between 1 and 10, it is recommended to supplement with assessment using other biosystems for validation. Weerapreeyakul et al. suggested a lower SI value (≥3) for the classification of a prospective anticancer sample [36].

When the SI index was calculated in our study, it was determined that the AgNPs-PTX compound was more effective in A549 cell lines than in other cancer cells (Table 3). The low toxicity of the compound in WI-38 normal cell lines is also a great advantage.

**Figure 5:**
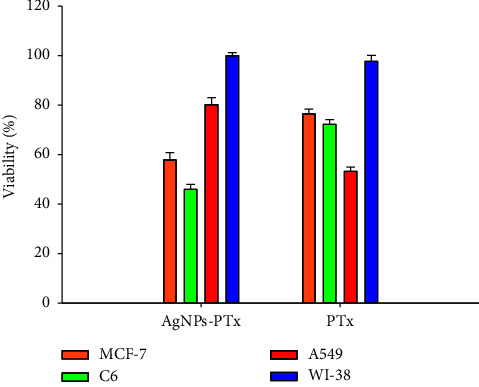
Percentage cell viability when AgNPs-PTX and PTX were applied to cells at a concentration of 10 *μ*g/mL (*n* = 6).

**Figure 6:**
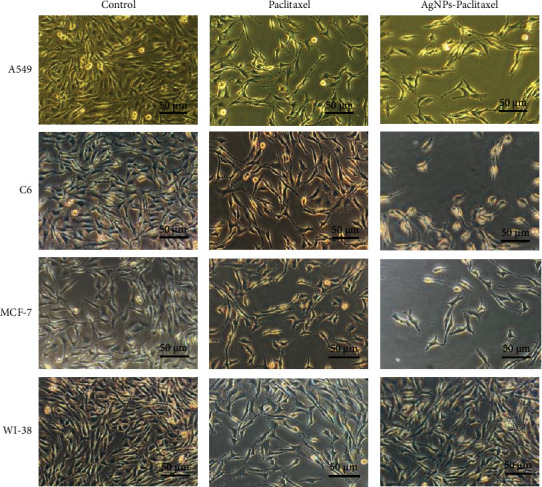
The observed cell morphology of A549, C6, MCF-7, and WI-38 cell lines after being treated for 24 h under a 100 Â inverted microscope.

In our study, three different (MCF-7, breast cancer; C6, glioma; A549, lung cancer) cell lines were used. AgNPs-PTX is more effective than PTX in all cell lines (Figure 5). In other words, its anticancer activity is higher and has low toxicity. AgNPs alone do not appear to be effective. It was observed that the entry of AgNPs-PTX into the cell was more effective. When the selectivity indexes are calculated, AgNPs-PTX appears to be more selective in the A549 cell line because the highest SI value was found in the A549 cell line. To obtain images of the cells used in the experiments, 15 *μ*g/mL AgNPs-PTX and PTX were applied to the cells, and images were taken with a ZEISS Axio inverted microscope (Figure 6).

Lung cancer is defined as a highly prevalent disease with an estimated 2.1 million cases in 2018. Due to this number of cases, lung cancer is the leading cause of cancer death in the world (18.5%). In short, lung cancer is a highly lethal disease and a serious health problem. There are two types of lung cancer: small-cell lung cancer (SCLC) and non-small-cell lung cancer (NSCLC) [20]. Among these, non-small-cell lung cancer accounts for 85% of all lung cancers. Because of this rate, NSCLC is the main source of tumor-related deaths globally, emphasizing the need for ever more effective treatment methods [5]. There are many scientific studies on nanodrug systems using nanotechnology in lung cancer treatment methods. In particular, many scientific studies on using silver nanoparticles as anticancer agents yield important and encouraging results [37]. In a study on A549 cells, the methotrexate drug delivery system (AgNPs-MTX) loaded on silver nanoparticles showed significant cytotoxic activity, which reduced the percentage of viable cells from 69% to 36% after 12 and 48 hours of incubation [38]. Akbarian et al. concluded that green synthesized ZnO nanocarriers loaded with paclitaxel in the MCF-7 cell line achieved PTX activity by minimizing their cytotoxic effects on normal cells (fibroblast) [39]. In a study with paclitaxel-loaded AgNPs, it was shown that the synthesized biomolecule has selective toxicity on A549 cells using ROS-mediated signaling pathways [40]. In another study, it was reported that green synthesized silver nanoparticles showed strong cytotoxic activity by reaching lower IC_50_ values on A549 and PC-3 cell lines [41]. Rudrappa et al. reported that P-AgNPs from *Plumeria alba* leaf extract showed a significant antiproliferative effect against the U118 MG (brain glioblastoma) cancer cell line [42].

### 3.3. Apoptosis Analysis

Annexin V/PI staining was used to determine the apoptosis values of A549 cells. Data on AgNPs-PTX and paclitaxel were evaluated by flow cytometry analysis (Table 4). In apoptotic cells, annexin V can serve as a probe for apoptotic cells due to its high affinity for the phospholipid phosphatidylserine (PS), which is displaced to the outside of the plasma membrane. Additionally, PI may be inserted between DNA strands in apoptotic cells and stain them. PI staining cannot spread across the membrane of living cells [43].

**Figure 7:**
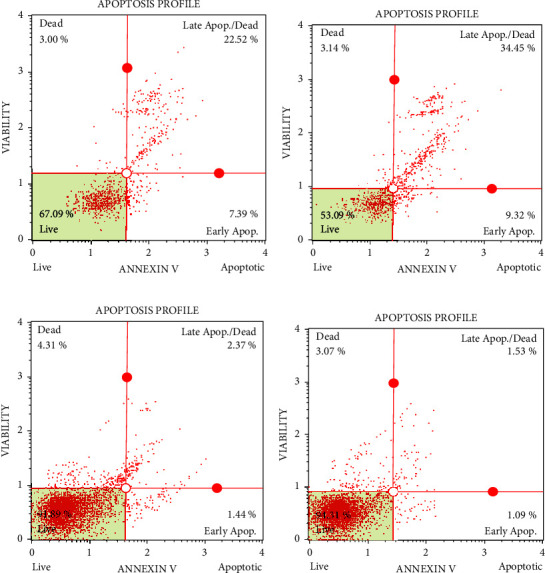
(a–d) Analysis of apoptosis of AgNPs, drug, and AgNPs-PTX on A549 and WI-38 cell lines by flow cytometry.

In cancer treatment, it is well known that the number of necrosis cells is supposed to be as low as possible, but the number of early and late apoptosis cells should be higher in proportion to the impact of the drug or applied chemicals on the cancer cells [37, 44]. The necrosis rates of PTX and AgNPs-PTX appear to be approximately close to each other (Figure 7). Therefore, it can be said that AgNPs-PTX did not make much change in the toxic effect. It is activated in the cell by using it only as a carrier mechanism. However, AgNPs-PTX appears to induce apoptosis. It has been determined that AgNPs-PTX induces DNA fragmentation and eventually increases apoptosis in cells compared to PTX. Moreover, the toxicity mechanism of apoptosis induced by AgNPs is well established in various cell lines such as human lung, ovarian, and breast cancer. Therefore, AgNPs are the optimal, suitable, and alternative nanoparticles that work with any anticancer drug [45]. In their study on green synthesized silver nanoparticles, Yuan et al. demonstrated the potential ability of silver nanoparticles to induce apoptosis in colon cancer cells as a result of experiments performed for apoptosis assessment [46]. Similarly, in a study with green synthesized silver nanoparticles, treatment of HCT-116 cells with C-AgNPs increased the number of early and late apoptotic cells compared with control (untreated) and C-AgNP-treated HCT-116 cells, and necrosis of these cells was also observed [43]. In studies with green synthesized silver nanoparticles, Liang et al. [47] and Venugopal et al. [48] observed that nanoparticles induced hyaluronic acid-induced apoptosis in cells through autophagy, mitochondrial dysfunction, cell cycle arrest, and lipid peroxidation. In their annexin V/PI apoptosis assay with synthesized polysaccharide silver nanoparticles, Chakraborty et al. reported that the change in the population of PC-3 live cells showed that the cell became apoptotic due to the cytotoxic activity of the biosynthesized PS-AgNPs [49].

### 3.4. Antimicrobial Activity

Antimicrobial activities of AgNPs and AgNPs-PTX on 8 bacteria and 2 yeast fungi were detected in the concentration range of 100 *μ*g/mL to 0.39 *μ*g/mL using the microdilution technique (MIC) (Table 5).

Among the 10 microorganisms tested, E. coli, K. pneumoniae, A. baumannii, P. aeruginosa, S. aureus, methicillin-resistant S. aureus (MRSA), and E. faecalis reported high antimicrobial activity according to the MIC ≤100 *μ*g/mL criterion, by reaching MIC values of 100 *μ*g/mL and below in both AgNPs and AgNPs‐PTX samples compared to reference sources. Both samples were found to be moderately effective against *B. cereus* bacteria and *C. albicans* and *C. tropicalis* yeasts according to 200 and above *μ*g/mL and 100 < MIC ≤ 625 *μ*g/mL criteria. When AgNPs and AgNPs-PTX were compared, it was determined that drug-added silver nanoparticles had higher antimicrobial activity than drug-free nanoparticles in all microorganism groups. When the MIC results of standard antibiotics were compared with AgNPs-PTX, it was determined that AgNPs-PTX indicated high antimicrobial activity against *A. baumannii, P. aeruginosa, S. aureus*, and *Methicillin-resistant S. aureus* (MRSA) bacteria [25].

AgNPs could be used as carriers or antimicrobial agents, as indicated by silver nanoparticles incorporated into membrane systems or microbicidal layers with antibacterial activity [5]. It has been shown in various studies that drug-loaded silver nanoparticles have antimicrobial activities. In the study by Ibraheem et al., AgNPs synthesized by chemical reduction technique were loaded with the antibiotic ciprofloxacin (CIP) to increase the antibacterial activity of CIP towards Gram-negative and Gram-positive bacteria. According to the results obtained, the synthesized nanocomposite AgNPs-PEG-CIP indicated higher antibacterial, antibiofilm, and antioxidant effects against the bacteria used in the assay compared to CIP alone [50]. In the study by Muenraya et al., Col-AgNPs were found to have higher activity than AgNPs and colistin against Gram-negative bacteria *(Escherichia coli, Klebsiella pneumoniae*, and *Pseudomonas aeruginosa*). According to their results, Col-AgNPs can increase antimicrobial activity and cell biocompatibility more than colistin and AgNPs [51]. In their in vitro studies with silver nanoparticles to investigate antimicrobial activity, Rafiq et al. reported that different concentrations of silver nanoparticles were effective against different microorganisms, such as bacteria (*E. coli, S. aureus, P. aeruginosa,* and *K. pneumoniae*) and fungi (*C*. *albicans*) [52]. AgNPs synthesized from aqueous leaf extracts of *Galphimia glauca* were found to have effective antimicrobial activity against *Pseudomonas aeruginosa* and *Candida glabrata* [53].

## 4. Conclusions

In our studies, the synthesized and characterized AgNPs-PTX indicated higher anticancer activity than paclitaxel in cancer cell lines (MCF-7, C6, and A549). In particular, a highly effective selective cytotoxic activity was detected in lung cancer cells (A549). The low toxicity in WI-38 healthy cells also demonstrates the success of the synthesized compound in minimizing side effects. It was also observed that AgNPs-PTX induced DNA fragmentation and eventually increased apoptosis cells compared to PTX. AgNPs-PTX synthesized in our study was tested in order to increase the antimicrobial activity of metal nanoparticles, which are preferred in research due to their microbicidal nature, by combining them with carriers. Antimicrobial activity was determined to be quite high, especially in Gram-negative microorganisms. Our results support the literature, and AgNPs-PTX has the capacity to provide a strong benefit in the treatment of non-small-cell lung cancer. It is a potential agent that can be used as a drug carrier, especially in lung cancer treatments. In further studies, the in vivo efficacy of this compound should also be tested. The detection of antimicrobial activity on certain microorganisms indicates that it can also be used as an antibacterial agent.

## Figures and Tables

**Table 1:** DLS results of the AgNPs and AgNPs-PTX.

Nano	Mean particle size (nm)	Particle size distribution (nm)	PDI	Zeta potential (mV)	Conductivity (mS/cm)
AgNPs	3.12	2.32–5.61	0.200	−47.3	0.314
AgNPs-PTX	34.56	24.36-58.77	0.535	−25.4	3.39

**Table 2:** IC_50_ results of the paclitaxel and AgNPs-PTX.

IC_50_ (*μ*g/mL)	MCF-7	A549	C6	WI-38
AgNPs-PTX	13.45 ± 1.05^*∗*^	8.58 ± 0.34^*∗*^	11.31 ± 0.97^*∗*^	56.07 ± 3.54
Paclitaxel	18.2 ± 2.09^*∗∗*^	16.49 ± 0.58^*∗∗*^	20.05 ± 1.02^*∗∗*^	41.65 ± 1.61
AgNPs	Not effective	Not effective	Not effective	Not effective

Standard deviation values are given as  ± Std, and all values are calculated as *n* = 3. ^*∗*^IC_50_: concentration that inhibited cell growth by 50%. ^*∗*^Statistically significant for AgNPs-PTX (*p* < 0.05). ^*∗∗*^Statistically significant for paclitaxel (*p* < 0.05).

**Table 3:** Index of SI.

IC_50_ (*μ*g/mL)	MCF-7	A549	C6
AgNPs-PTX	4.17	6.53	4.96
Paclitaxel	2.29	2.53	2.08

Standard deviation values are given as ± Std, and all values are calculated as *n* = 3.

**Table 4:** Percentage ranges of C6 cells after annexin V/PI.

Cells	Nanoparticles	Normal	Early apoptosis	Late apoptosis	Dead
A549	AgNPs-PTX	53.09 ± 0.34	9.32 ± 0.23	34.45 ± 1.02	3.14 ± 0.12
	PTX	67.09 ± 0.34	7.39 ± 0.66	22.52 ± 0.81	3.00 ± 0.04

WI-38	AgNPs-PTX	94.31 ± 2.02	1.09 ± 0.01	1.53 ± 0.02	3.07 ± 0.12
	PTX	91.89 ± 0.09	1.44 ± 0.02	2.37 ± 0.03	4.31 ± 0.05

Standard deviation values are given as ± Std, and all values are calculated as *n* = 3. AgNPs: silver nanoparticles and AgNPs-PTX: paclıtaxel-loaded silver nanoparticles.

**Table 5:** Antimicrobial activity results of the AgNPs and AgNPs-PTX.

Microorganisms (bacteria and yeasts)	MIC (*μ*g/mL) AgNPs	MIC (*μ*g/mL) AgNPs-PTX	MIC (*μ*g/mL) antibiotics	Antibiotics used
*Escherichia coli*	100	25	8	Amoxicillin
*Klebsiella pneumoniae*	100	50	16	Piperacillin/Tazobactam
*Acinetobacter baumannii*	100	50	64	Cefepime
*Pseudomonas aeruginosa*	25	12.5	16	TMP-STX
*Staphylococcus aureus*	50	25	64	Chloramphenicol
*Methicillin-resistant Staphylococcus aureus* (MRSA)	25	12.5	64	Ciprofloxacin
*Enterococcus faecalis*	50	12.5	8	Chloramphenicol
*Bacillus cereus*	>200	>200	1	Linezolid
*Candida albicans*	>200	>200	0.25	Fluconazole
*Candida tropicalis*	>200	>200	0.25	Fluconazole

The data obtained were studied as *n* = 3. Effective (MIC ≤ 100 *μ*g/mL), moderate (100 < MIC ≤ 625 *μ*g/mL), and weak (MIC > 625 *μ*g/mL).

## Data Availability

The data used to support the findings of this study are included in the article.

## References

[1] Alavi M., Kowalski R., Capasso R., Douglas Melo Coutinho H., Rose Alencar De Menezes I. (2022). Various novel strategies for functionalization of gold and silver nanoparticles to hinder drug-resistant bacteria and cancer cells. *Micro Nano Bio Aspects*.

[2] Krishnan P. D., Banas D., Durai R. D. (2020). Silver nanomaterials for wound dressing applications. *Pharmaceutics*.

[3] Xia Q. H., Ma Y. J., Wang J. W. (2016). Biosynthesis of silver nanoparticles using taxus yunnanensis callus and their antibacterial activity and cytotoxicity in human cancer cells. *Nanomaterials*.

[4] Gomes H. I., Martins C. S., Prior J. A. (2021). Silver nanoparticles as carriers of anticancer drugs for efficient target treatment of cancer cells. *Nanomaterials*.

[5] Sehgal S., Kumar J., Nishtha (2022). Involvement of gold and silver nanoparticles in lung cancer nanomedicines: a review. *Materials Today: Proceedings*.

[6] Siegel R. L., Miller K. D., Fuchs H. E., Jemal A. (2021). Cancer statistics. *Ca-A Cancer Journal for Clinicians*.

[7] National Institutes of Health (2023). Cancer of the lung and bronchus-cancer stat facts. https://seer.cancer.gov/statfacts/html/lungb.html.

[8] Mejía-Méndez J. L., López-Mena E. R., Sánchez-Arreola E. (2023). Activities against lung cancer of biosynthesized silver nanoparticles: a review. *Biomedicines*.

[9] Ferlay J., Soerjomataram I., Dikshit R. (2015). Cancer incidence and mortality worldwide: sources, methods and major patterns in GLOBOCAN 2012. *International Journal of Cancer*.

[10] Tao Y., Han J., Dou H. (2012). Paclitaxel-loaded tocopheryl succinate-conjugated chitosan oligosaccharide nanoparticles for synergistic chemotherapy. *Journal of Materials Chemistry*.

[11] Manivasagan P., Bharathiraja S., Bui N. Q., Lim I. G., Oh J. (2016). Paclitaxel-loaded chitosan oligosaccharide-stabilized gold nanoparticles as novel agents for drug delivery and photoacoustic imaging of cancer cells. *International Journal of Pharmaceutics*.

[12] Hu A., Guo J. Y., Alarifi H. (2010). Low temperature sintering of Ag nanoparticles for flexible electronics packaging. *Applied Physics Letters*.

[13] Peppercorn J., Herndon J., Kornblith A. B. (2005). Quality of life among patients with Stage II and III breast carcinoma randomized to receive high-dose chemotherapy with autologous bone marrow support or intermediate-dose chemotherapy: results from Cancer and Leukemia Group B 9066. *Cancer*.

[14] Da Silva C. G., Rueda F., Löwik C. W., Ossendorp F., Cruz L. J. (2016). Combinatorial prospects of nano-targeted chemoimmunotherapy. *Biomaterials*.

[15] Souza T. A., Franchi L. P., Rosa L. R., da Veiga M. A., Takahashi C. S. (2016). Cytotoxicity and genotoxicity of silver nanoparticles of different sizes in CHO-K1 and CHO-XRS5 cell lines. *Mutation Research/Genetic Toxicology and Environmental Mutagenesis*.

[16] Abdel-Fattah W. I., W Ali G. (2018). On the anti-cancer activities of silver nanoparticles. *Journal of Applied Biotechnology and Bioengineering*.

[17] Nandhini J. T., Ezhilarasan D., Rajeshkumar S. (2021). An ecofriendly synthesized gold nanoparticles induces cytotoxicity via apoptosis in HepG2 cells. *Environmental Toxicology*.

[18] Ameen F., Alown F., Al-Owaidi M. F. (2023). African plant-mediated biosynthesis of silver nanoparticles and evaluation of their toxicity, and antimicrobial activities. *South African Journal of Botany*.

[19] Hepokur C., Kariper İ. A., Mısır S. (2019). Silver nanoparticle/capecitabine for breast cancer cell treatment. *Toxicology in Vitro*.

[20] Panneerselvam C., Alalawy A. I., Albalawi K. (2022). Anticancer activity of bioactive compound chavicol as potential toxic against human lung cancer A549 cells. *Journal of Drug Delivery Science and Technology*.

[21] CLSI (2012). *(M07-A9) Methods for Dilution Antimicrobial Susceptibility Tests for Bacteria that Grow Aerobically*.

[22] CLSI (2012). *(M27-A2) Reference Method for Broth Dilution Antifungal Susceptibility Testing of Yeasts*.

[23] Kuete V. (2010). Potential of Cameroonian plants and derived products against microbial infections: a review. *Medical Plant*.

[24] Awouafack M. D., McGaw L. J., Gottfried S. (2013). Antimicrobial activity and cytotoxicity of the ethanol extract, fractions and eight compounds isolated from Eriosema robustum (Fabaceae). *BMC Complementary and Alternative Medicine*.

[25] The European Committee on Antimicrobial Susceptibility Testing (2023). Breakpoint tables for interpretation of MICs and zone diameters. http://www.eucast.org.

[26] Ivanova N., Gugleva V., Dobreva M., Pehlivanov I., Stefanov S., Andonova V. (2018). *Silver Nanoparticles as Multi-Functional Drug Delivery Systems*.

[27] Navya P. N., Kaphle A., Srinivas S. P., Bhargava S. K., Rotello V. M., Daima H. K. (2019). Current trends and challenges in cancer management and therapy using designer nanomaterials. *Nano convergence*.

[28] Hussein H. A., Abdullah M. A. (2022). Novel drug delivery systems based on silver nanoparticles, hyaluronic acid, lipid nanoparticles and liposomes for cancer treatment. *Applied Nanoscience*.

[29] Kesarwani P., Tekade R. K., Jain N. (2011). Spectrophotometric estimation of paclitaxel. *International Journal of Advances in Pharmaceutical Sciences*.

[30] Kaminari A., Nikoli E., Athanasopoulos A., Sakellis E., Sideratou Z., Tsiourvas D. (2021). Engineering mitochondriotropic carbon dots for targeting cancer cells. *Pharmaceuticals*.

[31] Umar Q., Huang Y., Nazeer A. (2022). Synthesis, characterization and anticancer activities of Zn 2+, Cu 2+, Co 2+ and Ni 2+ complexes involving chiral amino alcohols. *RSC Advances*.

[32] Gharibshahi L., Saion E., Gharibshahi E., Shaari A. H., Matori K. A. (2017). Influence of Poly (vinylpyrrolidone) concentration on properties of silver nanoparticles manufactured by modified thermal treatment method. *PLoS One*.

[33] Sahi S., Magill S., Ma L. (2018). Wavelength-shifting properties of luminescence nanoparticles for high energy particle detection and specific physics process observation. *Scientific Reports*.

[34] Shaik M. R., Khan M., Kuniyil M. (2018). Plant-extract-assisted green synthesis of silver nanoparticles using Origanum vulgare L. extract and their microbicidal activities. *Sustainability*.

[35] Danışman-Kalındemirtaş F., Kari̇per İ. A., Hepokur C., Erdem-Kuruca S. (2021). Selective cytotoxicity of paclitaxel bonded silver nanoparticle on different cancer cells. *Journal of Drug Delivery Science and Technology*.

[36] Weerapreeyakul N., Nonpunya A., Barusrux S., Thitimetharoch T., Sripanidkulchai B. (2012). Evaluation of the anticancer potential of six herbs against a hepatoma cell line. *Chinese Medicine*.

[37] Tunç T. (2024). Synthesis and characterization of silver nanoparticles loaded with carboplatin as a potential antimicrobial and cancer therapy. *Cancer Nanotechnology*.

[38] Rozalen M., Sánchez-Polo M., Fernández-Perales M., Widmann T. J., Rivera-Utrilla J. (2020). Synthesis of controlled-size silver nanoparticles for the administration of methotrexate drug and its activity in colon and lung cancer cells. *RSC Advances*.

[39] Akbarian M., Mahjoub S., Elahi S. M., Zabihi E., Tashakkorian H. (2020). Green synthesis, formulation and biological evaluation of a novel ZnO nanocarrier loaded with paclitaxel as drug delivery system on MCF-7 cell line. *Colloids and Surfaces B: Biointerfaces*.

[40] Zou J., Zhu B., Li Y. (2020). Functionalization of silver nanoparticles loaded with paclitaxel-induced A549 cells apoptosis through ROS-mediated signaling pathways. *Current Topics in Medicinal Chemistry*.

[41] Khedr A. I., Farrag A. F., Nasr A. M. (2022). Comparative estimation of the cytotoxic activity of different parts of cynara scolymus L.: crude extracts versus green synthesized silver nanoparticles with apoptotic investigation. *Pharmaceutics*.

[42] Rudrappa M., Rudayni H. A., Assiri R. A. (2022). Plumeria alba-mediated green synthesis of silver nanoparticles exhibits antimicrobial effect and anti-oncogenic activity against glioblastoma U118 MG cancer cell line. *Nanomaterials*.

[43] Azadpour A., Hajrasouliha S., Khaleghi S. (2022). Green synthesized-silver nanoparticles coated with targeted chitosan nanoparticles for smart drug delivery. *Journal of Drug Delivery Science and Technology*.

[44] Hassan M., Watari H., AbuAlmaaty A., Ohba Y., Sakuragi N. (2014). Apoptosis and molecular targeting therapy in cancer. *BioMed Research International*.

[45] Acharya D., Satapathy S., Somu P., Parida U. K., Mishra G. (2021). Apoptotic effect and anticancer activity of biosynthesized silver nanoparticles from marine algae Chaetomorpha linum extract against human colon cancer cell HCT-116. *Biological Trace Element Research*.

[46] Yuan Y. G., Zhang S., Hwang J. Y., Kong I. K. (2018). Silver nanoparticles potentiates cytotoxicity and apoptotic potential of camptothecin in human cervical cancer cells. *Oxidative Medicine and Cellular Longevity*.

[47] Liang J., Zeng F., Zhang M. (2015). Green synthesis of hyaluronic acid-based silver nanoparticles and their enhanced delivery to CD44+ cancer cells. *RSC Advances*.

[48] Venugopal K., Ahmad H., Manikandan E. (2017). The impact of anticancer activity upon Beta vulgaris extract mediated biosynthesized silver nanoparticles (ag-NPs) against human breast (MCF-7), lung (A549) and pharynx (Hep-2) cancer cell lines. *Journal of Photochemistry and Photobiology B: Biology*.

[49] Chakraborty B., Bhat M. P., Basavarajappa D. S. (2023). Biosynthesis and characterization of polysaccharide-capped silver nanoparticles from Acalypha indica L. and evaluation of their biological activities. *Environmental Research*.

[50] Ibraheem D. R., Hussein N. N., Sulaiman G. M., Mohammed H. A., Khan R. A., Al Rugaie O. (2022). Ciprofloxacin-loaded silver nanoparticles as potent nano-antibiotics against resistant pathogenic bacteria. *Nanomaterials*.

[51] Muenraya P., Sawatdee S., Srichana T., Atipairin A. (2022). Silver nanoparticles conjugated with colistin enhanced the antimicrobial activity against gram-negative bacteria. *Molecules*.

[52] Rafiq A., Tehseen S., Khan T. A. (2022). Biosynthesis of silver nanoparticles from novel Bischofia javanica plant loaded chitosan hydrogel: as antimicrobial and wound healing agent. *Biomass Conversion and Biorefinery*.

[53] Chakraborty B., Kumar R. S., Almansour A. I. (2021). Evaluation of antioxidant, antimicrobial and antiproliferative activity of silver nanoparticles derived from Galphimia glauca leaf extract. *Journal of King Saud University Science*.

